# Generation of a novel affibody molecule targeting *Chlamydia trachomatis* MOMP

**DOI:** 10.1007/s00253-021-11128-x

**Published:** 2021-02-01

**Authors:** Mingyang Li, Wei Shi, Jia Yang, Qi Wang, Haiyan Dong, Jun Chen, Lifang Zhang, Shanli Zhu

**Affiliations:** Institute of Molecular Virology and Immunology, Department of Microbiology and Immunology, School of Basic Medical Sciences, Wenzhou Medical University, Wenzhou, 325035 Zhejiang, People’s Republic of China

**Keywords:** *Chlamydia trachomatis*, MOMP, Affibody molecules

## Abstract

**Abstract:**

*Chlamydia trachomatis* (*C. trachomatis*) is the leading cause of preventable blindness worldwide and the most prevalent cause of bacterial sexually transmitted diseases. At present, there is no available vaccine, and recurrences after antibiotics treatment are substantial problems. Major outer membrane protein (MOMP) accounts for 60% of the outer mass of *C. trachomatis*, functioning as trimeric porin, and it is highly antigenic. Therefore, MOMP is the most promising candidate for vaccine developing and target therapy of *Chlamydia*. Affibody, a new class of affinity ligands derived from the Z-domain in the binding region of *Staphylococcus aureus* protein A, has been the focus of researchers as a viable alternative to antibodies. In this study, the MOMP-targeted affibody molecule (Z_MOMP_:461) was screened by phage-displayed peptide library. Further, the affinity and specificity were characterized by surface plasmon resonance (SPR) and Western blot. Immunofluorescence assay (IFA) indicated that the MOMP-binding affibody could recognize native MOMP in HeLa229 cells infected *C. trachomatis*. Immunoprecipitation assay confirmed further that Z_MOMP_:461 molecule specifically recognizes the epitope on relaxed trimer MOMP. Our findings provide strong evidence that affibody molecule (Z_MOMP_:461) serves as substitute for MOMP antibody for biological applications and has a great potential for delivering drugs for target therapy.

****Key points**:**

*• We screened a novel affibody molecule Z*_*MOMP*_*:461 targeting Chlamydia trachomatis MOMP.*

*• Z*_*MOMP*_*:461 recognizes the recombinant and native MOMP with high affinity and specificity.*

*• Z*_*MOMP*_*:461 could be internalized into live target cells.*

**Supplementary Information:**

The online version contains supplementary material available at 10.1007/s00253-021-11128-x.

## Introduction

*Chlamydia trachomatis* (*C. trachomatis*), an obligate intracellular bacterium, is the most common sexually transmitted bacterial pathogen worldwide (Centers for CDC 2015; Workowski and Bolan 2015). The majority of genital *Chlamydial* infection may result in severe complications such as pelvic inflammatory disease, ectopic pregnancy, and infertility if the infection is not treated (Lane and Decker 2016; Witkin et al. 2017). At present, no available vaccine and recurrence after antibiotics treatment are substantial problems.

*C. trachomatis* has a unique biphasic developmental cycle, which consists of two alternating cellular forms: the infectious, non-dividing elementary body (EB) and the proliferative, non-infectious reticulate body (RB) (Moulder 1991). Previous studies have shown that the cysteine-rich major outer membrane protein (MOMP) may function as a *Chlamydial* adhesin by promoting nonspecific interactions with host cells (Su et al. 1990; Mehlitz and Rudel 2013). Also, MOMP makes up 60% of the total outer membrane protein and is thought to play a role in maintaining structural integrity of the organism (Caldwell et al. 1981; Caldwell and Judd 1982) by forming a trimeric structure (Sun et al. 2007). Furthermore, during *Chlamydial* replication, MOMPs act as a porin for transporting ions and sugars across the outer membrane. The major *Chlamydial* membrane component harbors genus-, species-, and serotype-specific epitopes that elicit T cell responses and neutralizing antibodies (Baehr et al. 1988; Nunes et al. 2010). Thus, MOMP is regarded as a promising candidate for development of vaccine and novel therapeutics to treat *Chlamydia* infection.

Based on the scaffold of one of the IgG-binding domains of staphylococcal protein A, affibody molecules are small (6.5kDa), simple proteins composed of a three-helix bundle. The domain consists of 58 amino acids, and the binding surface has a randomized sequence of 13 amino acids to generate affibody libraries with a large number of ligand variants. Their ability to select and bind a given target protein with high affinity makes them an excellent affinity ligand (Nord et al. 1995). Affibody molecules as an alternative to monoclonal antibody (mAb) for biotechnological applications own to its unique advantages in screening, preparation, and practical application, such as (i) simple in vitro screening process and short cycle, (ii) high plasma clearance rate and strong tissue permeability in vivo, (iii)easy-to-label molecules (i.e., fluorescein and biotin) without affecting its affinity and producing nonspecific binding, and (iv)easy-to-improve thermostability, chemical stability, and recovery efficiency. Small size, high stability, and cost-effective production in bacteria make affibody molecules attractive for many medical and biological applications, including in vivo molecular imaging, receptor signal blocking, and protein detection (Frejd and Kim 2017;Ståhl et al. 2017).

In the present study, we describe the generation and characterization of a novel MOMP-binding affibody molecule for their ability to bind with recombinant and native MOMP and evaluated its usage in biological applications, such as Western blot, immunoprecipitation (IP), and immunofluorescence assay (IFA). Our data suggested that the MOMP-specific affibody molecules act as a novel probe for MOMP protein detection or a carrier to deliver effector molecules like toxin, drugs to develop novel therapeutics for *Chlamydial* infection.

## Materials and methods

### *C. trachomatis* infection

*C. trachomatis* strain E (ATCC VR-348B) was kindly provided by Professor Liu Yuanjun (Tianjin Medical University). HeLa229 cells (ATCC CCL-2.1) in 6-well plate were cultured at 37°C in a humidified atmosphere, containing 5% CO_2_ with RPMI 1640 (Gibco) medium supplemented with 10% (v/v) FBS. When HeLa229 cells grew and reached 80% confluence, the cells were washed with PBS (Gibco) and replaced with culture medium containing 30 μg/mL of DEAE-D (Sangon Biotech, Shanghai, China) for 30 min to increase the susceptibility of infection. During this period, *C. trachomatis* was pretreated by two freeze-thawing cycles, oscillation and centrifugation at 500×*g* for 5 min. The culture medium containing pretreated *C. trachomatis* was used to replace the original solution of cell culture plate incubated for 2 h. The multiplicity of infection (MOI) was 1 unless otherwise specified. After 48 h culture, the culture medium was removed, and the cells were washed twice with PBS and harvested in a sucrose-phosphate glutamic acid buffer (SPG) for storage. Some wells are fixed for Giemsa staining, immunofluorescence assays (IFA), and others for Western blot.

### Prepare MOMP for affibody screening

The nucleotide sequence of the gene encoding MOMP was retrieved from *C. trachomatis* strain E (GenBank accession no. DQ064286) and cloned into pET21a(+) for expression in *E.coli* BL21 (DE3). The 40 kDa product recognized by mouse His-tag mAb (MultiSciences Biothech Co. Ltd, China) (Fig. S1) was used for producing antiserum and affibody screening.

### Phage display screening

A combinatorial phage library was constructed, and phage selection of the binders to MOMP was performed in immuno tube according to the described method (Zhu et al.2020b; Xue et al.2016). Briefly, the thawed and diluted phage stock was infected at MOI = 20 with a helper phage M13K07 (Invitrogen), and the infected bacteria were incubated in a shaker at 37°C for 2 h. The cells were collected by centrifugation and resuspended in 2×YT containing ampicillin and kanamycin. Before harvesting, the culture was grown at 37°C overnight. Phage-containing cell lysate was clarified by centrifugation at 1000×*g* for 20 min. The lysate was filtered through 0.45 μm filter and concentrated by precipitation with 1/5th volume polyethylene glycol solution (20% PEG 8000, 2.5 M NaCl) for 45 min on ice. For phage extraction, the precipitated phagemids were centrifuged (20 min, 6000×*g*, 4°C) and resuspended in 2×YT culture. The target protein MOMP in carbonate coating buffer was coated on immuno tube (Greiner Bio-one, Germany) overnight at 4°C, and the unbound protein was removed by PBST (0.1% Tween20). After blocking with 5% non-fat milk in PBST for 1 h, the immuno tubes were incubated with phagemids at 37°C for 2 h. Subsequently, the tubes were washed six times with PBST. The bound phages were eluted with log phase *E. coli* TG1 cells at 37°C for 1 h without shaking. Then the *E. coli* TG1 culture was infected with M13K07 and subjected to next round of screening. In the last cycle, individual bacteria colonies were obtained, and the culture supernatant derived from the single colony was used for further ELISA screening.

### Screening ELISA

An ELISA-based assay was used to further verify their affinities to the target protein MOMP according to the methods described previously (Xue et al.2016). Briefly, the supernatants (100 μL) containing potential affibody molecules were loaded in microtiter wells, which had been previously coated with MOMP fusion protein. After blocking and washing, the plates were incubated with 100 μL of 1:15000 diluted mouse anti-M13 mAb (GE Healthcare, Piscataway, USA) per well for 1 h. After washing the wells four times, the plates were incubated with addition of 100 μL horseradish peroxidase (HRP)-conjugated goat anti-mouse IgG (1:5,000) per well for 1 h. The wells were washed four times, and 3,3′,5,5′-tetramethylbenzidine (TMB) solution was added to each well. After 30 min, stop solution (2 M H_2_SO_4_) was added, and the absorbance (OD) at 450 nm was measured in a Bio-tek ELISA microplate reader. The phages with relatively high signal of absorbance value, which bear potential affibody molecules with high affinity to MOMP, were selected for DNA sequencing and subsequently investigations.

### Affibody molecules production

After ELISA screening and DNA sequencing, the gene fragments encoding Z_MOMP_:461 (GenBank accession no. MT890566) affibody with high affinity were inserted into the pET21a (+) to produce the recombinant plasmids pET21a (+)/Z_MOMP_. To obtain affibody molecules, pET21a (+)/Z_MOMP_ were transformed into *E.coli* BL21 (DE3) and induced by IPTG for generating His-tag fusion proteins. The recombinant MOMP affibodies with a His-tag at the C-terminus were purified by chromatography with Ni-NTA agarose resin and verified by SDS-PAGE and Western blot with His-tag mAb. The purified proteins were further dialyzed in PBS using Slide-A-Lyzer according to the manufacturer’s recommendations. After determining concentration, the proteins were stored at −80°C for further use.

### Surface plasmon resonance analysis

To evaluate the target binding of the selected Z_MOMP_ affibodies to MOMP, surface plasmon resonance (SPR) was performed on a ProteOn XPR36 system (Bio-rad, CA, USA). The MOMP fusion protein (1 nM) served as the ligand was immobilized onto the surface of carboxylate glucans in HTG sensor chip (Bio-rad) and PBS was used as running buffer and for dilution of the analytes, as described previously (Zhu et al. 2020b; Xue et al.2016; Zhu et al. 2018). Subsequently, five or six concentrations of each affibody sample ranging from 125 to 4000 nM were prepared and injected over the chip surface to record sample binding to the surface. Z_WT_ affibody was set as a negative control. All experiments were carried out with a flow rate of 30 μL/min at 25 °C. The kinetic constants, including the association constant (ka), dissociation constant (kd), and affinity (KD, KD = kd/ka), were calculated by BIAcore T200 evaluation 3.0.2 software provided by the manufacturer, according to a 1:1 Langmuir binding model.

### Western blot

Infected HeLa229 cells were collected and lysed in RIPA lysis buffer supplemented with protease inhibitor (Beyotime Biotech Co. Ltd, China) and phosphatase inhibitor (Beyotime Biotech Co. Ltd, China) The proteins were separated by 12% SDS-PAGE and transferred to PVDF membrane, which was blocked with 5% skim milk in TBS buffer with 0.05% Tween20. The affibody Z_MOMP_:461 served as the primary antibody, mouse anti-His tag mAb served as the secondary antibody, and HRP-conjugated goat anti-mouse IgG (H+L) served as the third antibody. Rabbit MOMP antiserum was used as the positive control, and Z_WT_ affibody severed as the negative control. Glyceraldehyde 3-phosphate dehydrogenase (GAPDH) was used as an internal reference standard.

### Immunoprecipitation (IP)

Infected HeLa229 cells were collected and lysed as above. After centrifugation (12000 × *g* for 15 min at 4 °C), the cell lysate was mixed with rabbit MOMP antiserum at 4°C with gentle shaking; rabbit PBS antiserum severed as the negative control. After incubation overnight, protein A/G-conjugated agarose beads (Beyotime Biotech Co. Ltd, China) were added and incubated for an additional 3 h at 4°C. After centrifugation and multiple washes, the immunoprecipitated complex was mixed with SDS-PAGE loading buffer and boiled for 10 min. The sample was then separated by 12% SDS-PAGE and examined by Western blot analysis. Z_MOMP_:461 severed as primary antibody, and Z_WT_ affibody severed as the negative control. Heavy and light chains of immunoglobulin served as an internal reference standard.

Meanwhile, the cell lysate was mixed with Z_MOMP_:461 at 4°C with gentle shaking; Z_WT_ served as the negative control. After incubation overnight, mouse anti-His tag mAb and protein A/G-conjugated agarose beads were added and incubated for an additional 3 h at 4°C. After centrifugation and multiple washes, the immunoprecipitated complex was separated by SDS-PAGE and examined by Western blot analysis. MOMP antiserum served as primary antibody and PBS antiserum used as the negative control.

### Indirect immunofluorescence assay (IFA)

To determine whether the affibody molecule could bind to the MOMP native proteins, IFA was performed as previously described with minor modifications (Zhu et al. 2020a, 2020b). Briefly, HeLa229 (20,000 cells/well) was seeded into 24-well plate and incubated at 37°C for 24 h. When the cells were 80% confluence, the cells were infected with *C. trachomatis* according to the above methods. The original medium was exchanged for basic medium with 7.5 μM Z_MOMP_:461 or Z_WT_. After infection 24 h, a small number of inclusions are formed. After incubation for 6 h, cells were fixed with 4% paraformaldehyde at room temperature (RT) for 10 min. Subsequently, the cells were permeabilized by 0.2% saponin (Sigma Aldrich, Saint Louis, USA) at RT for 1h. After blocking, the cells were incubated respectively with mouse anti-His mAb and rabbit MOMP antiserum for analysis of affibody binding to native MOMP and co-location analysis of Z_MOMP_:461 and MOMP antibody. After washing, the binding was evaluated by the addition of secondary antibodies FITC-conjugated goat anti-rabbit IgG (H+L) (MultiSciences Biothech Co. Ltd, China) and Cy3-conjugated goat anti-mouse IgG (H+L) (Beyotime Biotech Co. Ltd, China) for 1 h. The nuclei of cells were counter stained with Hoechst33342 (Beyotime Biotech Co. Ltd, China) at RT for 5 min, and images were acquired by fluorescence microscopy (Leica TCS SP2 microscope).

## Results

### Identification of *C. trachomatis* infection

**Fig. 1:**
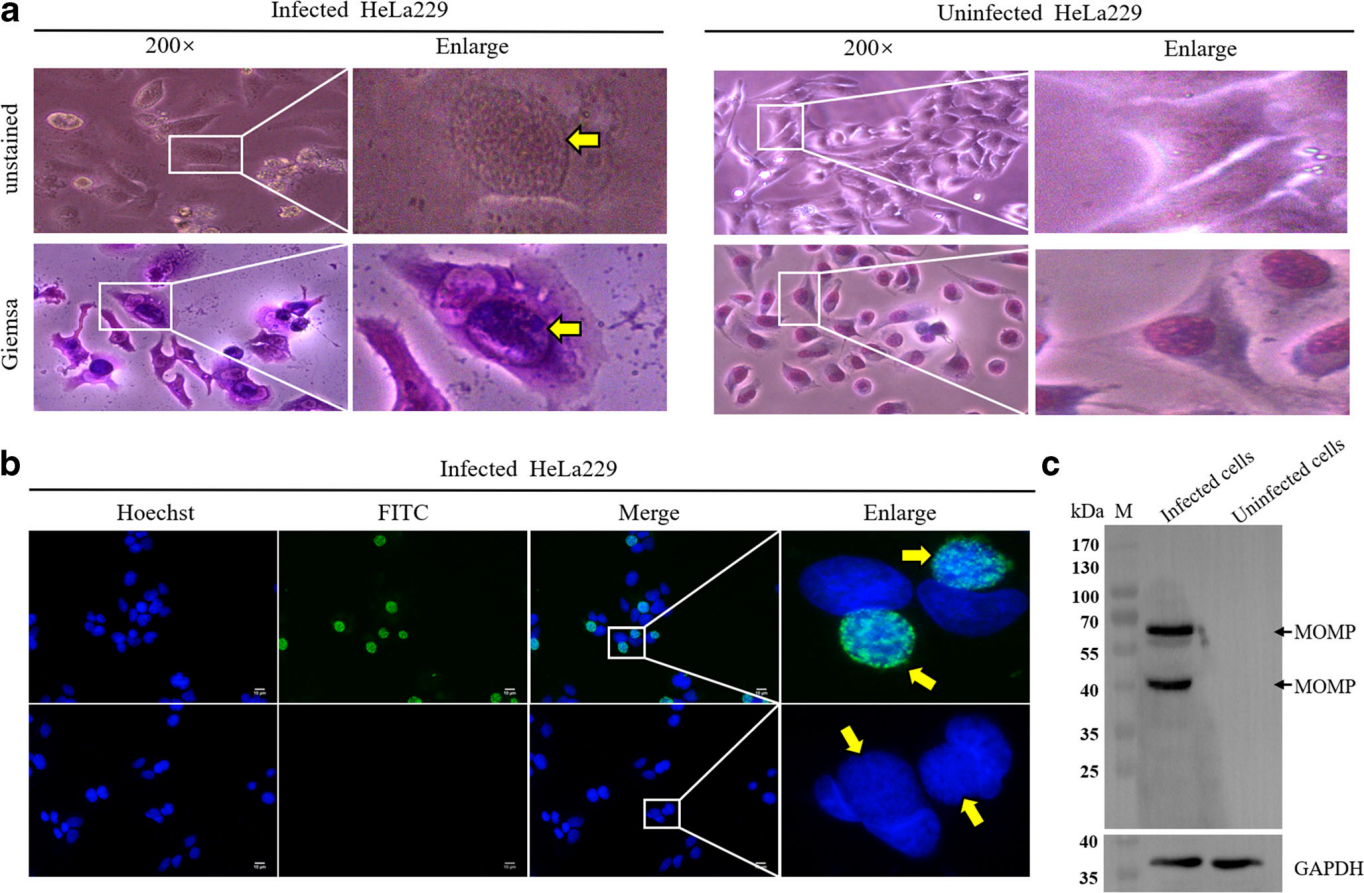
Identification of HeLa229 cells infected *C. trachomatis*. (**a**) Morphology of infected (left panel) and uninfected (right panel) HeLa229 cells. There were large particles of *C. trachomatis* inclusions (yellow arrows) in the cytoplasm of infected cells, and the nucleus was squeezed under light microscope. Giemsa staining showed that the inclusion body was darker than the nucleus, and small purple particles could be seen in the inclusion body. (**b**) The inclusion body (yellow arrows) in infected cells recognized by the antiserum against MOMP is shown in green, while nuclei of cells and *C. trachomatis* stained by Hoechst33342 are shown in blue. The merged images are shown cyan. RαMOMP, rabbit antibody against MOMP; RαPBS, rabbit antibody against PBS. (**c**) MOMP of infected cells was analyzed by Western blot. The polyclonal antibody against MOMP can recognize the compact trimer (67 kDa) and monomer (40 kDa) in infected cells

The HeLa229 cells infected *C. trachomatis* were fixed after 48 h post infection, and large granular *C. trachomatis* inclusions in the cytoplasm were observed under bright field microscopy (Fig. 1a). Giemsa staining revealed that the cytoplasmic inclusions, composed of *Chlamydial* organisms EB and RB, can be seen capping the nucleus, and a distinct space separates the inclusion body from the nuclear chromatin. As shown in Fig. 1b, the inclusions in the cytoplasm of infected cells could be simultaneously stained by MOMP antiserum and Hoechst33342, which indicated that our rabbit antibody could recognize MOMP in inclusion bodies of *C. trachomatis*.

Because of disulfide cross-link, the cysteine-rich monomer MOMP can form trimer protein. Published data have shown that there are two forms of MOMP trimers, compact and relaxed trimers (Sun et al. 2007; Feher et al. 2013). Both of the trimers are extracted from the native *C. trachomatis* EB, which suggested that they have different functions and immunity. In our present study, Western blot confirmed that MOMP antiserum can react with the target protein MOMP, about 67 kDa and 40 kDa, derived from the infected HeLa229 cells (Fig. 1c). The two bands are respectively compact trimers and monomers, which are consistent with previously published data (Sun et al. 2007). The above data indicated that MOMP antiserum prepared in-house could recognize specifically the compact trimer and monomer MOMP derived from infected HeLa229 cells and was used for subsequent investigations.

### Screening and selection of MOMP-binding affibody molecules

**Fig. 2:**
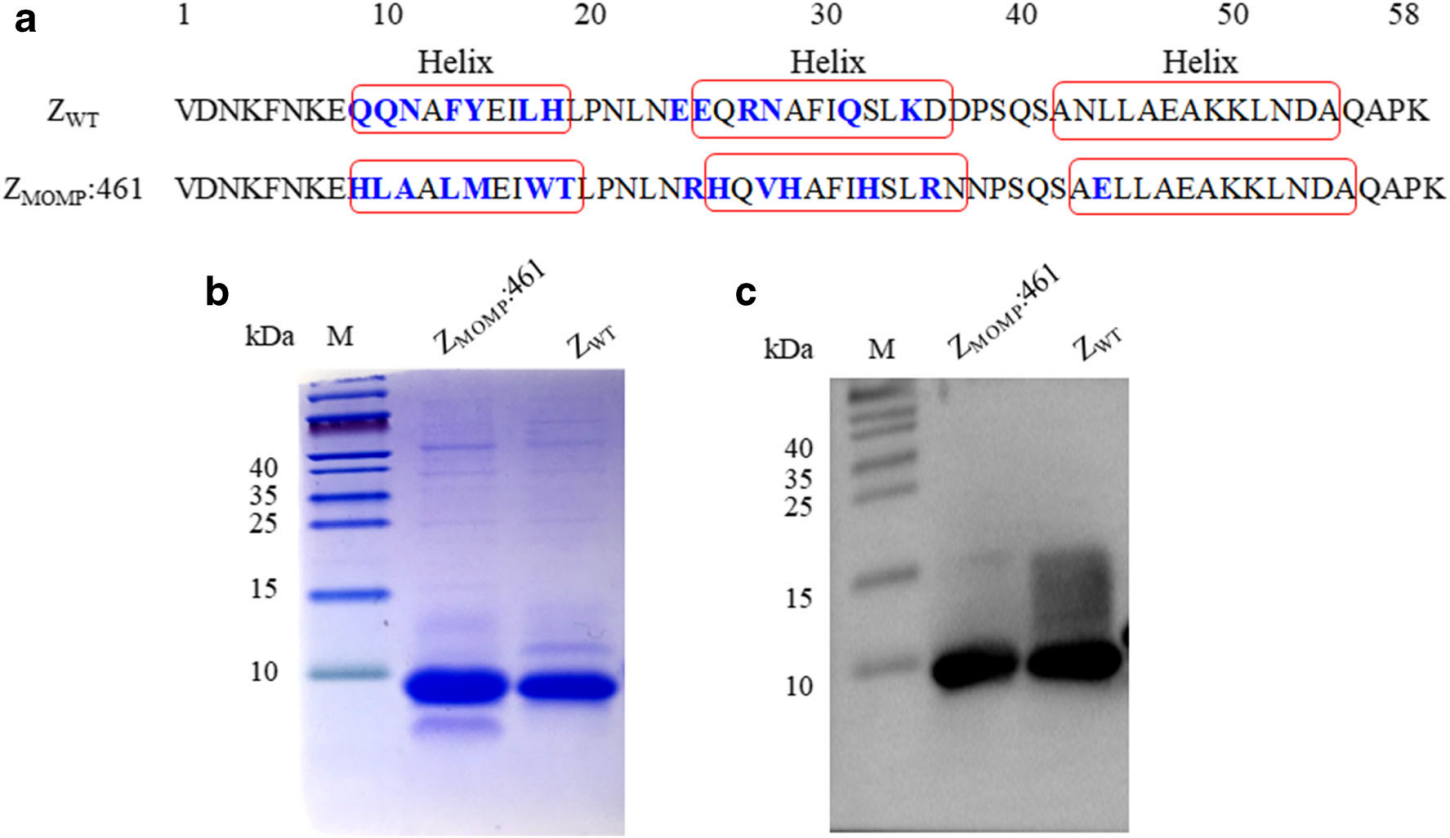
Z_MOMP_:461 affibody expression, identification, and purification. (**a**) Amino acid sequence alignment of Z_WT_ and Z_MOMP_ affibody. Thirteen randomized amino acid residues in Z_MOMP_ affibody are marked in blue and underlined. Red boxes indicate three-helical subdomains in the wild-type Z domain. (**b**) Total protein extracted from *E.coli* BL21(DE3) transformed with the recombinant expression plasmids and purified recombinant affibody protein Z_MOMP_:461 (Lane 1) and Z_WT_ (Lane 2) was separated by SDS-PAGE. (**c**) The purified Z_MOMP_:461 and Z_WT_ affibody were confirmed by Western blot with mouse anti-His tag as the primary antibody

A total of 480 clones were picked randomly for interaction with fusion MOMP with ELISA after three rounds of phage display library screening (Fig. S2), and 198 clones with high binding activity were pooled and submitted for DNA sequencing. Forty-seven clones possess very different sequences with 13 randomized amino acid residues in helices 1 and 2 of the Z domain when compared to the original affibody scaffold molecule Z_WT_. The framework region of affibody is highly homologous but highly diverse in the helix regions. One potential affibody molecule, Z_MOMP_:461, which showed the best binding to MOMP in ELISA screenings, was selected for further studies. The 13 randomized amino acid residues of Z_MOMP_:461 are presented in (Fig. 2a). The 174 bp gene fragments of the affibody were inserted into pET21a(+) vector in frame with a C-terminal His-tag. The expressed affibody proteins in *E. coli* were purified by Ni-NTA agarose resin. SDS-PAGE analysis showed that the molecular size is about 6.5 kDa (Fig. 2b), and Western blot analysis confirmed that the fusion proteins could be specifically recognized by His-tag mAbs (Fig. 2c). The final products were approximately in 95% of purity, which was used for subsequent investigations.

### The selected affibody bind to recombinant MOMP with high affinity

**Fig. 3:**
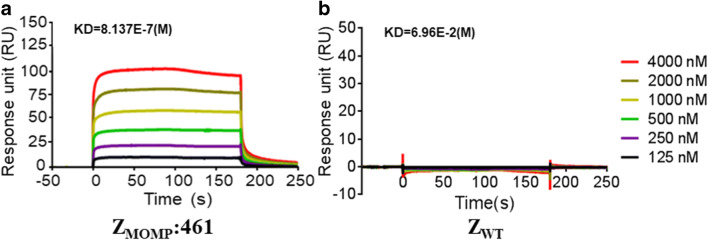
SPR analysis of the interaction between Z_MOMP_ and the target protein MOMP. Sensorgrams obtained after injection of the Z_MOMP_ affibody over a sensor chip containing purified recombinant MOMP. The binding abilities of different concentrations (from 125 to 4000 nM) of purified (**a**) Z_MOMP_:461 and (**b**) Z_WT_ to recombinant MOMP were tested using a SPR-based binding assay. Z_WT_ affibody was set as a control. Increasing concentrations of Z_MOMP_:461 led to dose-dependent increase of the response intensity, while Z_WT_ could not be detected in any effective interaction with MOMP fusion protein

After obtaining the purified Z_MOMP_:461, we employed surface plasmon resonance (SPR) biosensor assay to verify the binding affinity of the Z_MOMP_:461 to the target protein MOMP. The recombinant MOMP was immobilized on a sensor chip, Z_MOMP_:461, and control Z_WT_ affibody molecules were injected at different concentrations over the chip. Increasing concentrations of the affibody Z_MOMP_:461 led to dose-dependent increase of the response intensity with the concentration span 125~4000 nM, indicating that the affibody Z_MOMP_:461 binds directly to MOMP in a dose-dependent manner (Fig. 3a). In contrast, the molecule Z_WT_ could be detected in any effective interaction with MOMP fusion protein (Fig. 3b). Further kinetic BIAcore calculations revealed that the dissociation equilibrium constants (KD) of Z_MOMP_:461 were 8.137×10^-7^mol/L(Table S1), which was significantly lower than that of Z_WT_. These SPR data indicated that the selected affibody Z_MOMP_:461 binds to its target MOMP fusion protein with high affinity.

### The selected affibody binds to MOMP with high specificity

**Fig. 4:**
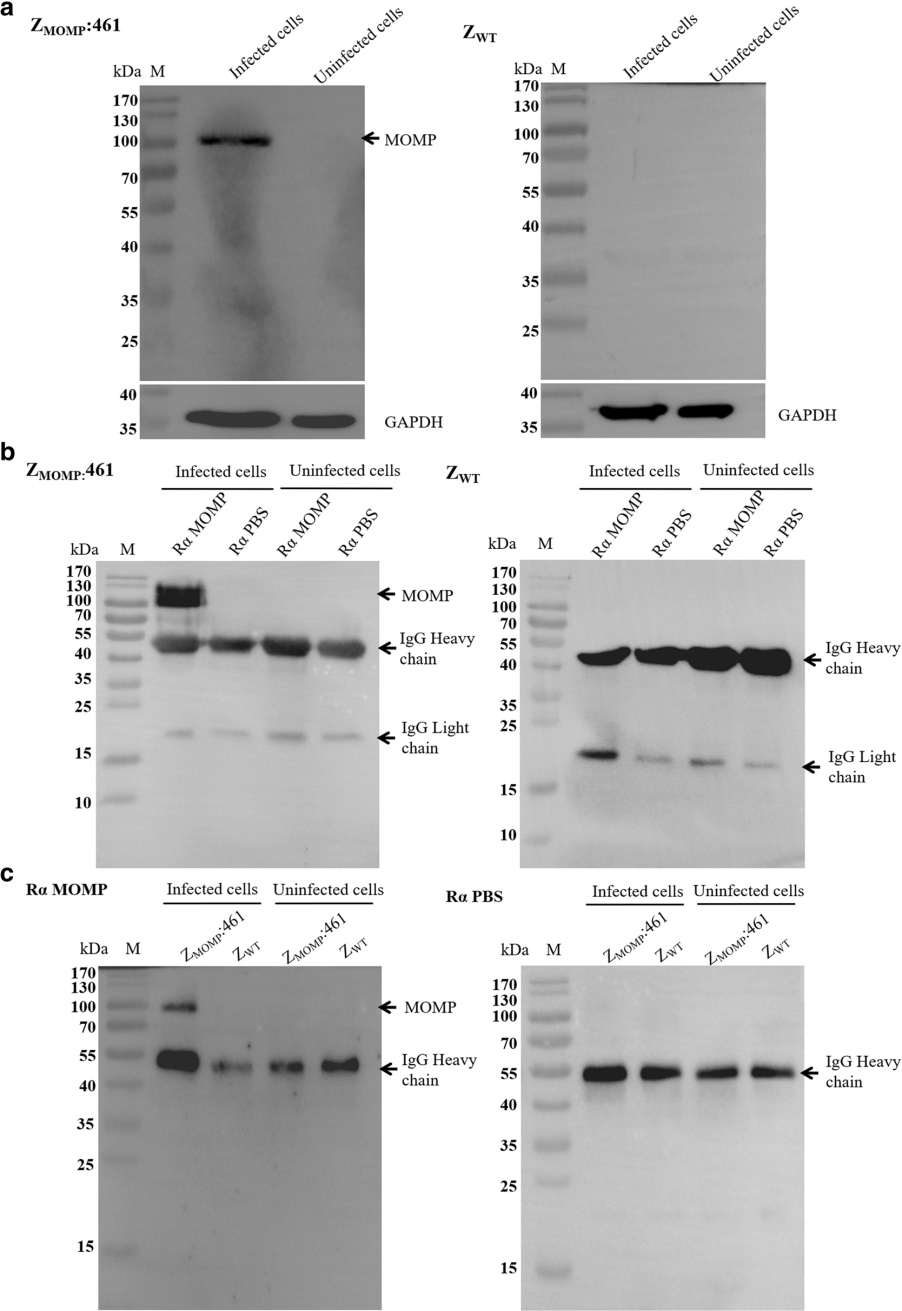
Identification of the specificity of Z_MOMP_ affibody to MOMP by Western blot and immunoprecipitation. The total protein of infected HeLa229 cells was separated by SDS-PAGE and analyzed by Western blot. (**a**) Western blot assay showed Z_MOMP_:461 recognized 100 kDa MOMP, while no band was observed in the Z_WT_ groups nor in uninfected groups. (**b**) The natural MOMP of infected cells precipitated by MOMP antiserum could be recognized by Z_MOMP_:461 at 100kDa; no band was observed in the Z_WT_ groups nor in uninfected groups. (**c**) The natural MOMP of infected cells precipitated by Z_MOMP_:461 could be recognized by MOMP antiserum at 100 kDa; no band was observed in the Z_WT_ groups nor in uninfected groups. The data confirmed that Z_MOMP_:461 bind specifically to target protein MOMP in infected cells. RαMOMP, rabbit antibody against MOMP; RαPBS, rabbit antibody against PBS

To verify further the specific binding of Z_MOMP_ to the target MOMP, the total protein of infected HeLa229 cells was analyzed by Western blot with Z_MOMP_:461 as the primary antibody. The results showed that Z_MOMP_:461 recognized 100 kDa MOMP, which is the relaxed trimer derived from the compact trimer treated with DTT (Fig. 4a), whereas no signal was observed in HeLa229 cells or in control affibody Z_WT_. The data indicated that the domain recognized by affibody Z_MOMP_:461 was mainly located in the relaxed trimer but rarely in the compact trimer or monomer MOMP.

IP was performed to confirm the specific binding of Z_MOMP_ to the target protein MOMP. The total protein of infected HeLa229 cells was mixed with the MOMP antiserum and precipitated with protein A/G agarose, subsequently analyzed by Western blot assay with Z_MOMP_:461 as the primary antibody. The results showed that Z_MOMP_:461 recognized the 100 kDa relaxed trimer MOMP (Fig. 4b), which indicated that the trimer MOMP precipitated by antiserum could be recognized by affibody Z_MOMP_:461. To confirm further the results, the cell lysate of infected HeLa229 cells was mixed with Z_MOMP_:461 firstly; after incubation of His-tag mAb and protein A+G agarose, the components were analyzed by Western blot with antiserum against MOMP as the primary antibody. The results showed that the 100 kDa relaxed trimer was observed as well (Fig. 4c). The above data confirmed that Z_MOMP_:461 affibody could bind target protein MOMP with high specificity.

### The selected affibody interact with native MOMP in HeLa229 cells infected *C. trachomatis*

**Fig. 5:**
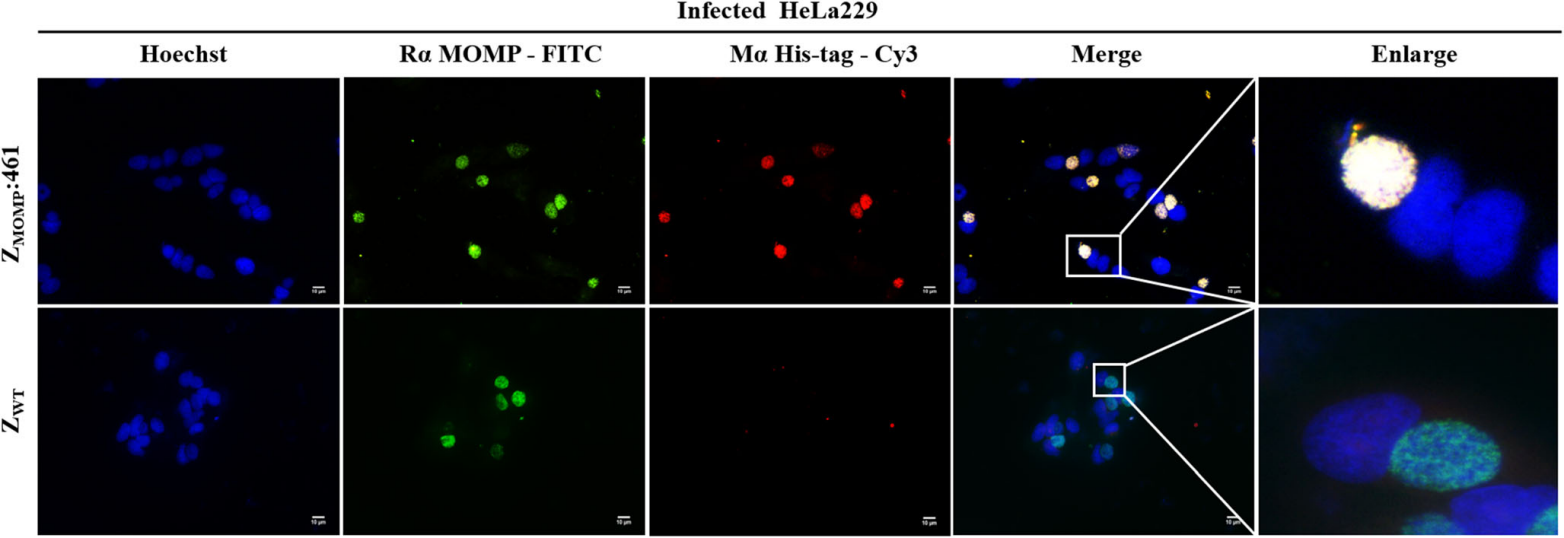
Z_MOMP_:461 affibody and rabbit anti-MOMP serum recognized the same native MOMP in infected HeLa229 cells. The infected HeLa229 cells were incubated with Z_MOMP_:461 affibody for 6 h. After fixation, permeabilization and blocking, the cells were incubated with mouse anti-His tag mAb and rabbit anti-MOMP serum. Z_MOMP_:461 affibody (red) and rabbit anti-MOMP serum (FITC) co-staining of native MOMP expressed in the cytoplasm of infected HeLa229. The nuclei of cells and *C. trachomatis* were stained with Hoechst 33342 (blue). The merged images showed the MOMP-specific co-staining (combined red, green, and blue to white), while infected cells incubated with Z_WT_ affibody are shown cyan (combined green and blue). Scale bar = 10 μm

Given that the affibody molecules selected in this study were able to bind to recombinant MOMP in SPR biosensor analysis and DTT-treated MOMP in Western blot and IP analysis, we next investigated whether the selected MOMP-binding affibody could also bind to native MOMP in infected HeLa229 cells using IFA. As shown in Fig. 5, the cytoplasmic inclusions can be recognized simultaneously by rabbit antiserum against MOMP (bright green fluorescence), MOMP-binding antibody (bright red fluorescence), and Hoechst33342 (blue), and the merged images showed the MOMP-specific co-staining (combined green, red, and blue to white).

As expected, there was no visible red staining in the cells incubated with the Z_WT_ control. Importantly, the live infected HeLa229 cells were incubated with Z_MOMP_:461 and Z_WT_ for 6 h before fixation and permeabilization, while the infected HeLa229 cells were incubated with antibody against MOMP after fixation and permeabilization. Thus, the above data indicated that Z_MOMP:_461 could be internalized into live cells and did specifically bind to native intracellular target protein MOMP in infected HeLa229 cells.

## Discussion

The success of target therapy in certain tumors has inspired people to extend this technology to infectious diseases. Currently, many conjugated mAbs are used to deliver varying effector molecules like drugs, toxins, and radionuclides specifically to infected cells (Cai and Berger 2011; Mariathasan and Tan 2017; Kapelski et al. 2015) and provided opportunity for developing novel therapeutics to treat infectious diseases. mAbs have become irreplaceable for diagnosis of infectious and chronic disease as well as targeted therapy; however, the use of conventional antibodies for targeted therapy is limited owing to the relatively large size, which impedes tissues penetration. As an alternative to antibodies, affibody molecules are very small in size (58 aa) and hence have favorable properties for diagnostic and as tumor ligands for drug delivery. To date, over 400 published studies show that affibody molecules have been selected for targeting more than 40 different proteins and served as affinity moieties in a variety of applications (Ståhl et al. 2017). The affibody-targeted proteins include epidermal growth factor receptor (EGFR)(Andersson et al. 2016; Oroujeni et al. 2018), human epidermal growth factor receptor 2 (HER2)(Orlova et al. 2006; Sörensen et al. 2016), human epidermal growth factor receptor 3 (HER3)(Malm et al. 2013; Schardt et al. 2017), EBV LMP1 (Zhu et al. 2020a) and LMP2 (Zhu et al.2020b), and human papillomavirus type 16 E7 (HPV16E7)(Xue et al. 2016; Zhu et al. 2018) and HPV18E7 (Wang et al. 2019).

At present, no available vaccine and recurrence after antibiotics treatment are substantial problems for *C. trachomatis* infection. Thus, it is urgent to carry out new therapy for the pathogen infection. MOMP is the most abundant surface-exposed protein of both EBs and RBs (Hatch 1996) and it has alternative conformations that may adapt to specific *Chlamydial* growth stages. Structural characterization of the MOMP remains elusive for several reasons. Recombinant MOMP protein has been expressed in different systems but has been intractable due to inclusion body formation and very low refolding yields, most likely due to its highly hydrophobic nature and presence of a high number or cysteine residues (Sun et al. 2007; Feher et al. 2013). MOMP comprises 370–393 amino acids with a high number of cysteine residues, which are involved in the formation of disulfide cross-linkages. The cysteine residues of MOMP are thought to not only form intramolecular bonds but also form intermolecular bonds stabilizing the MOMP trimer. Published data have confirmed that there are two types of MOMP trimers, compact and relaxed trimers (Sun et al.2007), and both are extracted from the native EB. After treated with DTT, loss of disulfide bond connection forces the compact trimer to the relaxed one. Analysis by 10% SDS-PAGE yielded two different bands with molecular mass of 67 kDa and 100 kDa, which correspond to compact and relaxed trimer, respectively.

Rabbit antiserum against MOMP (prepared-in-house) could specifically recognize the native MOMP expressed in inclusions containing EB and RB in the cytoplasm of infected cells. The recognition was further confirmed by Western blot, which showed that the rabbit antiserum against MOMP reacted with the MOMP extracted from the infected HeLa229 cells and two bands corresponding to the compact trimer (67 kDa) and monomer (40 kDa) were observed. Taken together, the above data demonstrated that the antiserum against MOMP prepared in-house could recognize mainly the epitopes of compact trimer and monomer MOMP and be used for subsequent investigation.

Affibody molecules play a vital role in the diagnosis and therapy of tumor and virus infectious diseases because of its small size, easy preparation, and strong penetration. At present, many affibody molecules targeting different proteins have previously been used successfully in many different biological applications, such as flow cytometry, immunofluorescence, immunochemistry, ELISA, IP, and so on and some, such as anti-HER2 affibody, anti-EGFR affibody, have been commercialized by Abcam company (www.abcam.com). Based on the high affinity and specificity of its interaction with the target protein, affibody molecule can be used as targeting vehicles for the delivery of a cytotoxic payload for cancer. Several preclinical studies have been performed to evaluate the concept, and thus far, investigations have focused on HER2-targeting affibody molecules (Zielinski et al. 2011), HPV16E7-targeting affibody molecules (Jiang et al. 2018), and EBV LMP-2 targeting affibody molecules (Zhu et al. 2020b). In addition to preclinical studies, some of them have entered phase 2 clinical trials for treatment. For example, Swedish affibody company (www.affibody.se) has developed an innovative autoimmune drug, ABY-035, which can specifically bind two subunits of IL-17A and albumin in serum. Phase II clinical trials of Psoriasis, ABY-035 showed excellent efficacy and safety (Identifier: NCT03591887). In this study, E serovar *C. trachomatis* MOMP served as the targets for affibody screening from the phage display library. The affinity of the selected affibody molecules in binding to recombinant MOMP was confirmed by in vitro SPR biosensor assays. Native MOMP extracted from the infected HeLa229 cells, treated with DTT, was analyzed by Western Blot with Z_MOMP_:461 as the primary Ab; the results showed that Z_MOMP_:461 recognized mainly 100 kDa MOMP (on relaxed trimer), which suggested that Z_MOMP_:461 obtained by screening with fusion MOMP could specifically bind to the domain in relaxed trimer MOMP. We speculated that the domains recognized by Z_MOMP_:461 are distinct from the epitope recognized by antiserum against MOMP.

To confirm the possibility that the Z_MOMP_:461 affibody specific recognizes MOMP, the native MOMP extracted from the infected HeLa229 cells was analyzed by IP assay. The cell lysate containing native MOMP was mixed with antiserum against MOMP and protein A+G, the precipitated components were analyzed by Western blot with Z_MOMP_:461 as primary antibody. Only one band corresponding to relaxed trimer (100 kDa) was observed, which indicated that the components pulled down by MOMP antiserum could be recognized by Z_MOMP_:461. That is to say, the MOMP compact trimer pulled down by antiserum switched to relaxed trimer after denaturation by heat and DTT, and the epitope in relaxed MOMP trimer was recognized by Z_MOMP_:461. To confirm the results, the cell lysate of infected HeLa229 cells was mixed with Z_MOMP_:461, mouse His-tag mAb, and protein A+G; the precipitated components were analyzed by Western blot with MOMP antiserum as the primary antibody. Similar results as above, one bond (100 kDa) corresponding to relaxed trimer was observed. The data demonstrated that the components pulled down by Z_MOMP_:461 could be recognized by rabbit antiserum against MOMP. We speculated that both compact and relaxed trimers are present in the lysate of infected HeLa229 cells, and Z_MOMP_:461 and antibody against MOMP recognized different domains located in different forms of trimer. Moreover, IFA co-staining confirmed that the MOMP antiserum and Z_MOMP_:461 are colocalization in infected HeLa229 cells, and both of them could recognize the native MOMP. Owing to the small size, affibody has been reported to be internalized into live cells and exhibited strong specific binding to native intracellular target protein (Zhu et al. 2020a). In our present study, the live infected HeLa229 cells were incubated with Z_MOMP_:461, and the IFA confirmed that Z_MOMP_:461 could be taken up by the target cells and bind to native MOMP in infected HeLa229 cells, which suggested that MOMP-binding affibody could act as a carrier to deliver effector molecules like drug or toxin to infected cells.

Overall, for the first time, we have produced and characterized a MOMP-specific affibody, Z_MOMP_:461, with high affinity and specificity. Z_MOMP_:461 could be internalized into live target cells; therefore, this affibody may serve as substitute for MOMP antibody in biological applications and have great potential for delivering drugs for target therapy.

## Supplementary Informations

ESM 1 (PDF 619 kb)

## Data Availability

The authors declare that the data supporting the findings of this study are available within the article and its supplementary information files.
