# Supplementary material for: Generation of a novel affibody molecule targeting Chlamydia trachomatis MOMP
Source: Appl Microbiol Biotechnol. 2021 Feb 1;105(4):1477–87. doi: 10.1007/s00253-021-11128-x (PMC7880956; doi:10.1007/s00253-021-11128-x)
Supplement: Supplementary file 1 — (PDF 619 kb) [file 253_2021_11128_MOESM1_ESM.pdf]

Supplementary materials

Applied Microbiology and Biotechnology

Title:

Generation of a novel affibody molecule targeting *Chlamydia trachomatis* MOMP

Authors:

Mingyang Li, Wei Shi, Jia Yang, Haiyan DONG, Jun Chen, Lifang Zhang, Shanli Zhu*

Affiliations:

Institute of Molecular Virology and Immunology, Department of Microbiology and Immunology, School of Basic Medical Sciences, Wenzhou Medical University, Wenzhou 325035, Zhejiang, People's Republic of China

Running title:

Affibody molecules targeting *Chlamydia trachomatis* MOMP

Keywords: *Chlamydia trachomatis*, MOMP, Affibody molecules

Corresponding authors:

*E-mail: sw852@126.com; tel: +86-577-86689910, Fax: +86-577-86689961

z

Table.S1 Kinetic Data from the SPR Biosensor Analysis of the Affibody Molecules

	Ka(1/Ms)	Kd(1/s)	KD(M)
Z_{MOMP}:461	4.357E+4	0.03545	8.137E-7
Z_{WT}	3.584E-2	0.002495	6.96E-2

Abbreviations: Ka, Association rate constant; Kd, Dissociation rate constant; KD, Dissociation equilibrium constant.

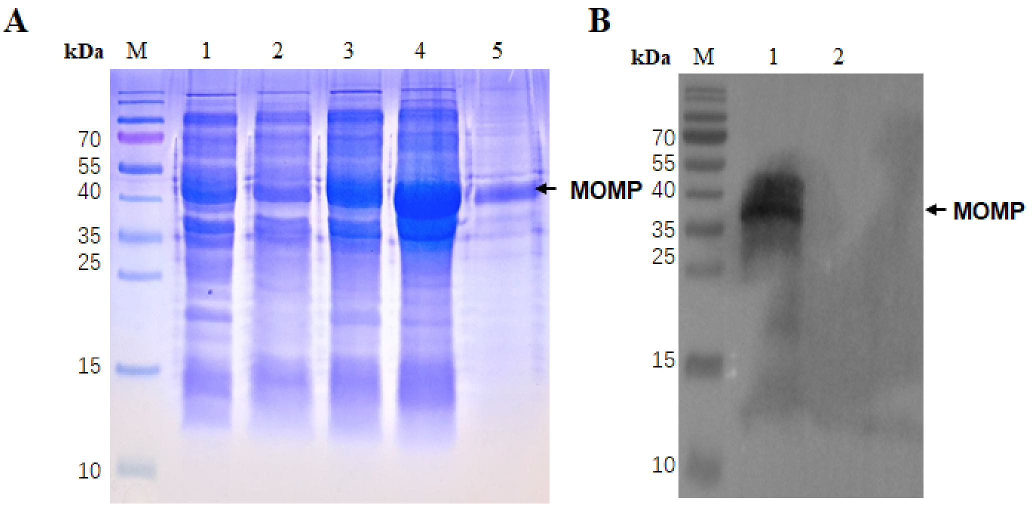

Figure.S1. SDS-PAGE and Western blot analysis of MOMP fusion protein.

(A) His-tagged MOMP fusion protein in Coomassie blue staining. M, protein ladder; 1. *E.coli* BL21(DE3); 2. *E.coli* BL21(DE3) transformed with pET21a(+) vector; 3. *E.coli* BL21(DE3) transformed with pET21a(+)/MOMP without IPTG induction; 4. *E.coli* BL21(DE3) transformed with pET21a(+)/MOMP with IPTG induction; 5.Purified His-tagged MOMP. **(B)** Western blot analysis of MOMP by His-tag mAb. M, protein ladder;1. *E.coli* BL21(DE3) transformed with pET21a(+)/MOMP with IPTG induction; 2. *E.coli* BL21(DE3) transformed with pET21a(+) with IPTG induction.

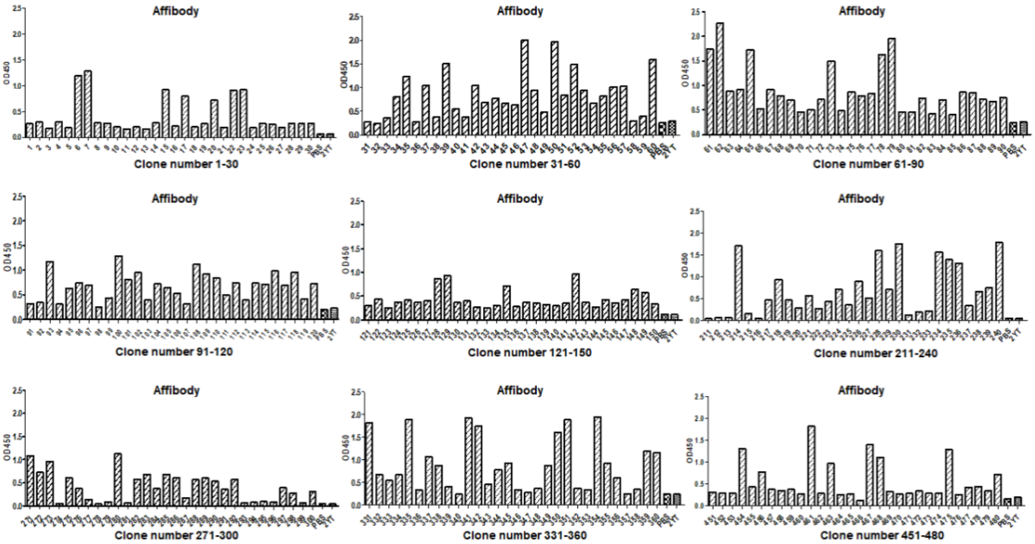

Figure.S2. A representative ELISA screening for target-binding activity of MOMP affibody.

The supernatants containing potential affibody molecules were loaded in microtiter wells, which had been previously coated with fusion MOMP. A total 480 clones from phage library were screened for its interaction with MOMP by an ELISA assay and high signal intensity clones were selected for DNA sequencing.
